# Supplementary material for: Egalitarian norms can deflate identity-bias link in real-life groups
Source: PLoS One. 2025 Aug 28;20(8):e0330484. doi: 10.1371/journal.pone.0330484 (PMC12393728; doi:10.1371/journal.pone.0330484)
Supplement: Attachment — Submitted filename: Review Letter.docx [file pone.0330484.s002.docx]

**Response Letter**

Dear Editor,

We are deeply grateful to you and the reviewers for the insightful and constructive feedback on our paper. We believe that these helped us immensely in improving the manuscript. We reorganized the introduction, method, and discussion sections according to reviewers’ feedback. We made numerous additions to emphasize the context and social identity background of the paper’s rationale. We tried to focus on the purpose of the paper with new headings. We reformulated our hypotheses to address the problems related to the hypotheses raised by both reviewers. We rewrote the entire text according to these changes. We have re-numbered each comment and provided our answers below each. We indicated the changes in red ink. We hope that this version of the paper is suitable for the journal.

Reviewer #1

1. At least two issues seem crucial for the novelty of the research program, but are undertheorized. First, given that a lot of attention is paid to the importance of focusing on egalitarian norms instead of fairness norms, authors should elaborate more on the theoretical distinction between fairness and egalitarianism and, if existent, present research showing different outcomes between fairness and egalitarianism social norms, ideologies, and so on.

**Response:** We thank Reviewer 1 for their suggestion to further elaborate on the theoretical distinction between fairness and egalitarianism and to incorporate research demonstrating different outcomes associated with these social norms. While there is no empirical work to the best of our knowledge comparing the effects of fairness and egalitarianism norms, we believe that our existing rationale provides a strong foundation for this distinction, particularly in explaining why fairness may not always translate to equal treatment, and how the egalitarian norm offers a more consistent and neutral framework for resource allocation. Specifically, we highlight that fairness is a more self-serving and context-dependent principle, encompassing multiple distributive norms that may or may not include concerns around equality, group status and group needs; whereas egalitarianism is more narrowly defined as a commitment to strict equality in outcomes, showing less sensitivity to differences in status or needs.

That said, we agree that it needs further conceptual clarification. We have now expanded our explanation with resources demonstrating the theoretical distinction, notably through the context-dependent nature of fairness that heavily limits the external validity of earlier findings. We believe these additions enhance the necessity of this distinction and provide a clearer rationale for our focus on egalitarian norms. Hence, we revised the related content under the new heading of *Fairness vs. Egalitarianism* on pages 7-8 as follows:

The way we operationalize the ingroup norm manipulation differs from Jetten and colleagues [14], who examined norms under fairness and favouritism conditions. However, a key issue with fairness is that it does not inherently promote equality between groups. In fact, fairness may paradoxically reinforce ingroup favouritism when it is perceived as a means to rectify past injustices [31]. Favouring the ingroup can be interpreted as a fair strategy under zero-sum conditions as well, given that even non-zero-sum games are associated with a fiercer competition over resources than they actually are [32]; after all, resource allocation tasks are by default a zero-sum game. Research also suggests that fairness judgments may be anchored in proportionality rather than strict equality [54]. That is, individuals tend to assess fairness based on the relative contributions of parties involved rather than an egalitarian principle that mandates equal division, leading to outcomes where inequality is perceived as fair.

Another factor that can shape fairness perceptions is status concerns, as higher-status group members are more likely to see unequal outcomes as fair when they believe their group is entitled to greater rewards [60]. This highlights that fairness can be self-serving as it enables high-status groups to justify and maintain inequalities while presenting them as fair [33]. Even in the absence of clear status differences, people still apply fairness flexibly to justify unequal distributions. For instance, in ultimatum game scenarios, individuals tend to offer unequal distributions while believing their offers are fair, despite the fact that recipients consider these allocations as unfair [61]. This finding demonstrates how fairness norms can be misaligned between groups, further reinforcing the idea that fairness can be self-serving and does not inherently promote equality.

Given that fairness is an ambiguous concept subject to multiple interpretations -including compensatory justice, proportionality, and status maintenance- it is challenging to ensure that fairness norms consistently lead to non-favouring distributions. This conceptual ambiguity has led researchers to replace fairness with egalitarianism as a more consistent, neutral, unambiguous and non-retaliatory framework for resource allocation [13]. By emphasizing strict equality in outcomes, egalitarian norms rule out context-dependent interpretations of fairness, which can be endorsed to justify ingroup favouritism. Thus, our study chooses the norm of egalitarianism over fairness to avoid the unintended biases that fairness-based distributions can easily introduce. By focusing on an unequivocally equal allocation of resources, we ensure that fairness, when interpreted in a way that permits ingroup favouritism via status reinforcement or retaliatory motives, does not undermine the impartiality of our norm manipulation.

We have also added the following paragraph on pages 26-27 to emphasize that this area is open to future research, noting the lack of studies directly comparing the effects of fairness and egalitarianism across different social contexts.

Third, we acknowledge that further empirical studies are needed to systematically compare the effects of fairness and egalitarianism norms across different intergroup settings. Future research should explore whether fairness norms lead to different intergroup outcomes compared to egalitarianism, especially in competitive versus cooperative contexts.

2. Second, I would encourage the authors to explain more thoroughly why they think that relationships between fans of different football teams are not conflictual. News often reports clashes between rival fans, and this appears to have happened recently also in Turkey. Therefore, I do not find convincing the argument that the authors have considered a non-conflictual intergroup context.

**Response:** We thank Reviewer 1 for their valuable feedback regarding the classification of our research context as non-conflictual. We understand the concern that football rivalries can, at times, escalate into conflicts, and we appreciate the opportunity to clarify our rationale for considering our study context as competitive rather than conflictual.

To address this, we have revised our manuscript to provide a more explicit distinction between competitive and conflictual intergroup settings. In particular, we now highlight how our study differs from prior research on intergroup bias in conflictual contexts (e.g., Jetten et al., 1997; Çoksan & Cingöz-Ulu, 2022), where participants directly allocated resources between two opposing groups (e.g., Kurds vs. Turks). In contrast, in our study, participants allocate resources to their own team, with the remaining funds being distributed among all other teams in the league. Given that their team is not in direct and realistic competition with the majority of these teams, this allocation process does not align with the characteristics of intergroup conflict.

We have also expanded our discussion under the newly added section “Sampling Real-life Group Members in a Competitive Context” to further elaborate on why we do not classify this setting as conflictual. Moreover, we have incorporated an additional limitation acknowledging that certain subsets of football fans experience heightened tensions and that future research may explore how ingroup norms function in highly conflictual fan interactions.

These revisions ensure a clearer articulation of our reasoning and contextual framework. We appreciate the reviewer’s insightful comment, which has helped refine our discussion of the competitive nature of our study context.

Hence, we created the *Sampling Real-life Group Members* heading and revised the relevant contents on page 6 as follows:

We sampled non-WEIRD real-life group members (football team fans) contrary to many studies in the social identity approach; thus, we aim to extend the findings on the association between ingroup identification and ingroup bias to real and competitive groups in a non-conflictual context. In this way, we thought that we could both increase the generalizability of the findings and contribute to the understanding of cultural dynamics by filling this sampling gap in the literature and thus providing valuable information for practical applications.

Unlike previous studies that examined intergroup bias in conflictual settings [13-14], where resource allocation occurred directly between two competing groups (e.g., Kurds vs. Turks), our study did not position participants in a direct intergroup competitive framework. Instead, participants allocated resources to their own group while the remaining funds were distributed among all teams in the league. Given that their team is not in realistic competition with the majority of these teams, this allocation process lacks the hallmarks of intergroup conflict. While competition among football teams exists, it does not constitute a conflictual intergroup setting in the way that ethnic or political rivalries do. To reiterate, in our study, participants do not allocate resources between their team and a direct rival, but rather between their team and the general league, which consists of many teams with whom their team has no meaningful rivalry. This distinction is crucial in understanding why we do not classify this as a conflictual intergroup context.

Moreover, in Türkiye, being a football fan is a very strong and consistently prominent form of social identity. Fan identity plays an important social role in shaping social relations and group loyalties not only during matches but also in daily life such as reading about the matches and buying licensed merchandise [29]. For many individuals, loyalty to soccer teams creates a lifelong sense of belonging, and this identity is particularly strong among fans of major teams [30]. Therefore, the identity of football fans in Türkiye provides a relevant and powerful context for the study of group norms and identification processes.

3. Furthermore please note that, on page 13, I would expect an explanation about the differential effects of descriptive and injunctive norms. Otherwise, the claim that injunctive norms might be more relevant to group identity than descriptive norms seems not well justified.

**Response:** We thank Reviewer 1 for their comment regarding the need for further justification of why injunctive norms might be more relevant to group identity than descriptive norms.

We acknowledge that the original discussion on page 13 required a clearer distinction between these two types of norms (*On a related note, please also see our response to point 21 raised by Reviewer 2*). We have expanded our explanation by emphasizing that injunctive norms–defined as the moral or socially endorsed *ought to* rules of behavior–play a fundamental role in defining and reinforcing group behaviors. Unlike descriptive norms, which simply reflect what people *do*, injunctive norms signal the collective values and moral expectations of a group. Since individuals are motivated to conform to these norms to maintain their social identity and affiliation, injunctive norms exert a stronger influence on group-relevant outcomes. The emphasis on injunctive, rather than descriptive, norms, however, is only important insofar as we want it to influence group-level outcomes.

This contention is supported by prior research demonstrating that when individuals perceive a strong injunctive norm within their ingroup, they experience greater normative pressure to align their behavior accordingly, as failing to do so may threaten their standing within the group (e.g., Hogg & Reid, 2006; Cialdini et al., 1991). We have now made this theoretical connection more explicit in the revised manuscript. Additions made read as follows on page 16:

To investigate this, we employed a novel methodology of manipulating ingroup norms through a group discussion. While our research question did not concern the differential effects of descriptive and injunctive norms, tapping into the latter was necessary for norm manipulation. This is because injunctive norms, representing what ought to be done, exert a significant and consistent influence on emotional and behavioural responses toward rival outgroups, whereas the effects of descriptive norms, representing common behaviours, are less reliable [63]. Therefore, injunctive norms might be more relevant to group identity than descriptive ones [49]. When individuals perceive a strong injunctive norm within their ingroup, they experience greater normative pressure to align their behaviour accordingly, as failing to do so may threaten their standing within the group [39, 64]. This distinction is crucial, as the belief that people should allocate resources equally between ingroup and outgroup members is deemed as an injunctive norm rather than a descriptive one [65]. This means that while people may endorse intergroup equality as the morally right thing to do, they do not necessarily expect it to be widely practised. This approach aligns with the conceptualization of injunctive norms in earlier work [64, 66], where social expectations are communicated through consensus and perceived obligation to the ingroup rather than mere behavioural trends observed within the ingroup**.**

Finally, we had written an additional paragraph indicating when people would and would not conform to descriptive norms, and another paragraph making the relationship between group identity and injunctive norms more explicit, but we removed those two paragraphs because that version of the article would have been problematic in terms of word count. However, we would like to inform you that we are willing to add them if Reviewer 1 asks us to do so.

4. The authors have summarized literature suggesting that social norms are also formed and negotiated via social interactions in group discussions, emphasizing the active role of each group member. While I find the arguments convincing, and at the same time I have appreciated the novel and creative experimental manipulation of social norms, built on classic research, I think there is a mismatch between the theoretical rationale and the actual experimental manipulation. Indeed, there were no real group discussions, and therefore there was no negotiation. Each participants was merely invited to express their opinion after being exposed to the opinion of three other alleged ingroup members. I would encourage the authors to carefully think about the best justification for their method, and to avoid overemphasizing some strengths which might not fully apply to their method.

**Response:** We thank Reviewer 1 for their thoughtful feedback and for recognizing both the strength of our theoretical rationale and the novel nature of our experimental manipulation. We appreciate your reservations around the alignment between our theoretical framework and experimental manipulation. By rights, we acknowledge that our manipulation did not involve a group discussion in which participants actively negotiated norms through *back-and-forth* interactions. However, we maintain that our approach still captures a key mechanism of social norm formation–norm transmission via group consensus and social exposure–which aligns with established research on how norms emerge and exert influence within groups (Cialdini et al., 1991; Hogg & Reid, 2006; Moscovici, 1974).

Unfortunately, standardizing the manipulation required us to avoid back-and-forth norm negotiation as we could not have ensured consistent and applicable responses to the wide range of participant input. While such direct negotiation was not part of our design, we nevertheless argue that the perception of a collective consensus among ingroup members is a central mechanism through which ingroup norms take hold. Research shows that group norms are often inferred from perceived group unanimity (Turner, 1991; Bicchieri, 2006). Our methodology effectively simulated this process by ensuring that participants were exposed to a structured, sequential presentation of the opinions of ingroup members each addressing the one who spoke prior to them, thereby creating the perception of a normative expectation. Furthermore, by having participants respond after observing all other ingroup members endorse a particular stance, our design showed that individuals may conform to perceived group norms without direct negotiation (Abrams et al., 1990).

On the other hand, we acknowledge that our methodology does not fully replicate the interactive nature of group discussions. However, the absence of direct negotiation does not undermine the effectiveness of our manipulation, as norms can be internalized through perceived group consensus without explicit back-and-forth dialogue. Our design prioritizes methodological consistency and internal validity by ensuring that all participants are exposed to the same normative statements in a controlled manner, eliminating variability that could arise from unscripted group interactions.

That said, we acknowledge the importance of avoiding any unintended overstatement regarding the interactive nature of our paradigm. To address this concern, we have refined our discussion to clarify that while our manipulation does not involve back-and-forth negotiation, it effectively models norm transmission through perceived group dialogue, which is a widely recognized pathway for norm adoption (Please see our response to point 3 and 23 on a related note). We sincerely appreciate this constructive feedback, as it has allowed us to more clearly articulate the strengths of our approach while acknowledging this important limitation on norm negotiation. The addition reads as follows on pages 25-26:

On the other hand, we would like to draw attention to the limitations of our research in various aspects. An important limitation is that our manipulation did not include direct group discussion in the form of back-and-forth norm negotiation. However, it still captures a key mechanism of norm formation, via group consensus and social exposure, consistent with earlier work [37, 39, 64]. Standardizing the manipulation required avoiding a back-and-forth interaction, as ensuring standard responses across various inputs from participants would be unfeasible. Nevertheless, our design effectively simulated norm formation by structuring sequential ingroup member statements, and thus fostering the perception of a normative expectation, more closely aligning with injunctive than descriptive norms [65]. Although group identification and perceived group consensus may be sufficient to infer and act on norms even in the absence of direct negotiation [67], future research may incorporate AI-driven, adaptive chat interfaces where participants engage in dynamic back-and-forth discussions with programmed ingroup members. This would allow for a standardized but interactive norm negotiation process, addressing the limitation of the current design while maintaining experimental control.

5. The organization of the introduction is not optimal. I would suggest to first summarize relevant literature, and then state specific hypotheses which should be based on literature.

**Response:** While addressing the above feedback, we grouped the topics under certain headings. Also, to address this feedback, in parallel with the feedback, we first gave a summary of the entire literature. Then, we stated our hypotheses.

6. As a minor related issue, I find it surprising to first have the hypotheses about the interaction effects and then the hypotheses of the main effects. The reverse order sounds more logical.

**Response:** We thank the reviewer for pointing out this counterintuitive ordering. The primary reason for this ordering is that the novel aspect of our research is primarily related to the hypothesis concerning the interaction effect. The topics covered by the main effects (the effects of identification and norm) have already been examined in different contexts in the literature. However, investigating what happens when identification and ingroup norms point in different directions in terms of ingroup bias, as in our study, presents a more unique contribution. Even so, we acknowledge that the suggested revision may be a more reasonable approach. On the other hand, Reviewer 2 specifically recommended that we conceptualize interaction effects as our hypotheses. Additionally, after considering Reviewer 1’s feedback regarding the reporting of hypotheses and the corresponding analyses, we recognize the need to present this section more clearly and appropriately. Therefore, in line with the suggestion, we have revised the paragraph on hypotheses on page 19 as follows:

Based on this literature, throughout the two studies in the current research, we hypothesized that in the presence of a significant interaction between ingroup identification and ingroup norm, when the ingroup norm is favouritism-oriented, participants will show greater ingroup bias as their ingroup identification increases (H1), whereas when the ingroup norm points at egalitarianism, participants will show less ingroup bias as their ingroup identification increases (H2). All data and measurements of both studies are provided at the OSF link of the paper, as follows <https://osf.io/2fha7/?view_only=81ae7ad0bada483ebc344da3d40ed6b8>.

7. Most importantly, the section “current research” is extremely long and not very focused. For example, the first page of “current research" should be part of the theoretical summary. Most of the paragraphs are about the conceptualization of social norms (favouring the ingroup vs. egalitarian) and on the novel experimental manipulation of social norms. However, in “current research” I would expect paragraphs explaining and justifying the whole research program, and not a focus mainly on the second study. Overall, the organization of the whole introduction (from the very first page to “current research”) should be improved.

**Response:** We thank Reviewer 1 for this feedback. To address comments 5 and 6, we have reevaluated the overall structure of the manuscript. We added various new headings to emphasize key topics and then shortened the Current Research section to make it more focused. In its revised form, this section now briefly reiterates the core arguments behind our hypotheses before explicitly stating them. Then, we once more emphasize a key expectation regarding ingroup norms. Finally, we conclude the section by directing readers to the page where we present the datasets and measurement tools used in the studies. We hope that these revisions align with Reviewer 1’s expectations.

8. The discussion of Study 1 is partly confusing. Authors state “Moreover, high identifiers showed higher levels of ingroup bias under the favouritism norm (H1 & H2).” However, H1 and H2 are presented as hypothesis about the effects of ingroup identification for those who perceive norms as favouring the ingroup (H1) or egalitarian (H2) and not as a single hypothesis that egalitarian norms buffer the effect of ingroup identification. Then H2 is not confirmed, and the above-mentioned sentence is very imprecise.

**Response:** As stated in our responses to the feedback above, we have revised the ordering of the hypotheses. In parallel with this revision, we have also modified the first paragraph of the Discussion section in Study 1 as follows:

This cross-sectional study showed that ingroup identification positively predicted ingroup bias while ingroup norms unexpectedly did not. The link between identification and ingroup bias is strengthened when the ingroup norm is favouritism (H1), while the significance of this link disappears in the presence of an egalitarian ingroup norm (H2); however, our initial expectation was that under an egalitarian ingroup norm, the relationship between identification and ingroup bias would be negative. Theoretically speaking, it was plausible to expect in light of the relevant literature that higher identification would predict lower ingroup bias under the egalitarian ingroup norm. However, this hypothesis was not supported [13].

Additionally, we have adjusted the relevant sections under this heading in accordance with these changes.

9. The presentation of Study 2 findings is very confusing. Authors first present findings from the ANOVA, and this is fine given the 2 X 2 experimental design. It is also fine to me to decompose the interaction even if it was not significant. However, why decomposing the interaction also with social norms as predictor and ingroup identification as moderator (“Post-hoc comparisons between experimental conditions indicate that participants with favouritism ingroup norm show more ingroup bias (N = 30, M = 5.21, SE = .93) than those with egalitarianism norm condition (N = 31, M = 2.15, SE = .92; p = .021, 95% CI [.476, 5.645]) on low ingroup identification condition. Similarly, participants with favouritism ingroup norm show more ingroup bias (N = 32, M = 8.33, SE = .90) than those with egalitarianism norm condition (N = 41, M = 2.86, SE = .80; p < .001, 95% CI [3.092, 7.852]) on high ingroup identification condition.”)? Most importantly, the following section is extremely confusing. What have the authors exactly done? Have they tested the same regression analysis of Study 1 with the measured variables (and not with the experimental manipulations)? If yes, this needs to be very well specified. The sentence “Since 2 X 2 between-subjects ANOVA results indicate the ingroup norm’s moderating role, …” is not a sufficient justification. Importantly, it makes no sense to calculate power on the explained variance of the regression analysis with measured variables. The main novelty of Study 2 is the experimental design, therefore power needs to be calculated based on ANOVA findings (or alternatively on a regression analysis with the two experimental manipulations and their interaction as predictors).

**Response:** We are grateful to Reviewer 1 for this important feedback. Upon re-evaluating the text, we realized that we had reported the analysis results in a somewhat confusing manner. We fully agree with all the points raised. The main reason we conducted the ANOVA along with the moderation analysis was to see if we could replicate a similar pattern with different analyses, but we certainly acknowledge that this is not a standard method and can be seen as confusing. Therefore, considering our revised hypotheses, we have completely removed the ANOVA results from the manuscript in Study 2. In this version, we focus solely on the moderation analysis results and clearly state what they mean in relation to our hypotheses. Additionally, in line with this decision, we have reported power analyses for both studies.

10. The discussion of Study 2 is very confusing. First, the wording “high identifiers” and “low identifiers” should not be used if authors are referring to “participants in the high identification condition” and “participants in the low identification condition”.

**Response:** We thank Reviewer 1 for this feedback. We acknowledge that not using these terms in a standardized manner might have caused confusion. In line with the suggestion, we have revised all instances of "high/low identifiers" throughout the manuscript accordingly.

11. Second, the sentence “High identifiers were more strongly influenced by ingroup norm than low identifiers under the favouritism norm condition” is very imprecise. In both identification conditions, those in the favouritism norm condition reported higher intergroup bias than those in the egalitarian norm condition, and the interaction was not significant. The authors state “It is essential to note that the ingroup norm did not have a main effect on ingroup bias in Study 1, while it produced a potent effect in Study 2, especially on those in the high identification condition. This must be the direct result of the manipulation method exclusively designed for this study”. However, their justification is in my view not correct. Indeed, in Study 2 the main effect of social norms is detected also when analysing measured social norms (and not only experimentally manipulated social norms).

**Response:** We appreciate Reviewer 1’s feedback regarding the clarity of our discussion of Study 2. First of all, we acknowledge that the phrase 'High identifiers were more strongly influenced by ingroup norm than low identifiers under the favouritism norm condition' may have been imprecise. Also related to previous feedback, we have articulated this section more clearly. The heading *Discussion* as it stands now focuses solely on the moderation analysis results, as we noted in our response to feedback #9. We believe that this way we have simplified the text considerably and made it easier to follow. Moreover, we appreciate Reviewer 1’s feedback on our justification regarding the effect of norm manipulation. We acknowledge that our original phrasing may have overemphasized the role of the experimental manipulation in producing the norm effect. As the reviewer correctly points out, the effect of social norms was also present when analyzing measured ingroup norms, indicating that the norm effect is not solely attributable to our experimental design. We have revised the discussion to clarify that while our manipulation likely heightened norm salience, the observed effects also reflect naturally occurring variations in participants’ perceived ingroup norms. This clarification ensures that our conclusions more accurately represent the interplay between manipulated and naturally perceived norms. As a result, we have arranged the first two paragraphs under this heading as follows:

Study 2 extended the results of Study 1. In parallel with Study 1, ingroup identification positively predicted favouritism. But unlike Study 1, Study 2 revealed in this study we observed a main effect of the norm; participants showed more ingroup bias when they reported that ingroup norms were more indicative of favouritism. Moreover, under the favouritism norm condition, people with high ingroup identification were more strongly influenced by the ingroup norm than people with low ingroup identification. In short, under the favouritism norm condition, people with high ingroup identification showed more favouritism (H1), whereas when the ingroup norm indicated egalitarianism, as in the first study, the relationship between ingroup identification and ingroup bias lost statistical significance (H2).

It is essential to note that while the ingroup norm did not have a main effect on ingroup bias in Study 1, it exerted a strong influence in Study 2. This might suggest that our manipulation played a role in reinforcing the norm effect by making ingroup norms more salient. However, as the effect of social norms was also observed when analyzing measured norms, we recognize that norm salience likely contributed to the observed effects rather than being their sole cause. Intragroup discussions may prompt individuals to reflect on their place in the group and this enhances one’s commitment to their ingroup’s shared opinions [55]. As with Study 1, however, we failed to find evidence to support our expectation of a negative identification-ingroup bias association on egalitarianism norm condition. It seems to be the case that the challenges in extending egalitarian treatment to outgroups discussed in the first study were also upheld by the results of Study 2.

12. Authors should report in the main text a justification for the sample size of both Studies. Please note that in experimental study 2 there are less than 50 participants per cell, therefore the study might be underpowered (see also the fifth comment).

**Response:** As noted in our response to Feedback #9, we reported post-hoc power analyses for both studies. As we noted in the text, statistical power for both studies was above the minimum acceptable power, .80.

13. The discussion should be thoroughly revised after modifications are implemented to the introduction and to the presentation of Study 1 and 2 findings. Indeed, in the current version of the discussion, there are imprecisions. For example, the sentence “Moreover, the variance of ingroup bias explained by ingroup identification, ingroup norm, and their interaction is 11% in Study 1, while it is 42% in Study 2” should not be used as an illustration of the effectiveness of the social norms experimental manipulation, because it appears to refer to the regression analysis with measured variables in both studies. To make a proper comparison, authors should check the Study 2 variance explained in the ANOVA. As another example, on page 23, line 511, the authors discuss the role of “social interactions” in their social norms experimental manipulation. However, there were no social interactions, as participants expressed their opinion after being exposed to the opinion of alleged ingroup members.

**Response:** We thank Reviewer 1 for this feedback. In line with the previous comments, we have thoroughly reviewed and rewritten the entire *General Discussion* heading. In this revised version, we have ensured that the text aligns closely with the reported analysis results and hypotheses.

14. Finally, I would recommend the authors to carefully re-check the article before resubmission.

**Response:** We confirm that we had the manuscript proofread before resubmission.

In conclusion, we believe we have appropriately addressed all of Reviewer 1’s feedback. We hope that our work as it stands is suitable for the journal.

Reviewer #2:

15. The authors state the following: "*Throughout the two studies in the current research, we hypothesized that (H1) when the ingroup norm is favouritism, participants show more ingroup bias as ingroup identification increases, and (H2) when the ingroup norm is egalitarianism, participants show less ingroup bias as ingroup identification increases since highly identified group members are more inclined to conform to perceived ingroup norms. In terms of main effects, our expectation is that (H3) participants with high ingroup identification show more ingroup bias than participants with low ingroup identification, and (H4) those in the condition where the ingroup norm is favouritism show more ingroup bias than those in the condition where the ingroup norm is egalitarianism.*" Here, the second and the third hypothesis is at odds: If the ex-ante expectation is that high group identification leads to less ingroup bias if the group norm is egalitarian, then the main effect cannot be that ingroup bias is larger with high group identification -this will only be driven by the ingroup favoritism group norm.

**Response:** We thank the reviewer for pointing out the inconsistency between H2 and H3. Upon revisiting our hypotheses, we acknowledge that H3 should not be considered a general main effect but rather a pattern that emerges under the ingroup favoritism norm condition. Furthermore, we realized that we should evaluate the hypothesis concerning the ingroup norm in a similar manner. A similar critique was also raised by Reviewer 1. Therefore, we have revised our hypotheses to explicitly state that the effect of ingroup identification on ingroup bias is contingent on the ingroup norm condition. Specifically, we now clarify that higher ingroup identification leads to increased ingroup bias only when the ingroup norm is favoritism. To ensure consistency, we have also reviewed our discussion and results sections to align with this clarification. We believe these revisions address the issue raised by the reviewer and enhance the clarity of our theoretical framework. The revised hypotheses can be found on page 10 as follows:

Based on this literature, throughout the two studies in the current research, we hypothesized that in the presence of a significant interaction between ingroup identification and ingroup norm, when the ingroup norm is favouritism-oriented, participants will show greater ingroup bias as their ingroup identification increases (H1), whereas when the ingroup norm points at egalitarianism, participants will show less ingroup bias as their ingroup identification increases (H2). All data and measurements of both studies are provided at the OSF link of the paper, as follows <https://osf.io/2fha7/?view_only=81ae7ad0bada483ebc344da3d40ed6b8>.

16. Subsequent paragraphs on page 5 explain how those with low group identification would be predicted to behave. Here, the writing is unclear, as this prediction only makes sense as a comparison to those with high group identification in the current study, not stand alone. For example, what would this predict for people with low group identification: "these members still tend to look to ingroup norms to determine the correct behaviour in the social environment because these contexts provide strong guidance to members on what they should do. Thus, even individuals with low ingroup identification still follow these strong social cues to determine appropriate behaviour in the social context, indicating a tendency to conform to group norms, though to a lesser extent than high identifiers" Does this mean that those with low group identification have low ingroup bias generally or only lower than those with high group identification. If the former, is it because they are a different type of person (for example, they may be generally less "groupy" as in recent work by Kranton) or is it because, they care less about their one particular group identity.

**Response:** We appreciate Reviewer 2’s detailed and insightful feedback on this section. Their concerns regarding the clarity of our discussion on how individuals with low ingroup identification behave in comparison to those with high ingroup identification prompted us to refine our explanations.To address this issue, we have revised the relevant section to clarify that individuals with low ingroup identification do not entirely reject ingroup norms or exhibit no ingroup bias. Instead, empirical evidence suggests that the relationship between ingroup identification and ingroup bias persists even at lower identification levels, though the effect is weaker. We explicitly state that individuals with low ingroup identification may still demonstrate ingroup bias but to a lesser extent than those with high identification. Moreover, we have elaborated on the reasons why individuals with low ingroup identification may still adhere to group norms, albeit to a lesser degree. In the revised text, we now discuss multiple external and situational factors that can influence norm adherence, including social pressure and fear of exclusion, practical benefits of norm adherence, maintaining future identity options, and cultural influences. By incorporating these explanations, we clarify that norm adherence among low-identifiers does not necessarily stem from strong intrinsic motivation but rather from strategic and pragmatic considerations.

Additionally, we have refined our wording to make it clear whether low-identifiers exhibit generally low ingroup bias or merely lower ingroup bias compared to high-identifiers. We now explicitly state that low-identifiers are still likely to exhibit some degree of ingroup bias, but their group identity plays a less central role in shaping their behaviour. Thus, they are less likely to favour their ingroup unconditionally and are more prone to adjusting their behaviour based on external social dynamics.

We believe these revisions resolve the ambiguity in the original manuscript and provide a clearer theoretical grounding for our discussion on low-identifiers. Final paragraph on pages 3-5 are as follows:

People with low ingroup identification tend to comply with group norms less than people with high ingroup identification. The weak bond these members have with the group may not sufficiently motivate them to exhibit behaviour in line with the norm because, for these members, conforming to group norms may not be as important or rewarding as for highly identified ingroup members [23]. Therefore, they are more likely not to feel obliged to adopt group norms, which may explain why low ingroup identification is associated with a lower tendency to conform to the ingroup norm [26-27]. However, this does not mean that people with low ingroup identification entirely reject group norms or that they show no ingroup bias. In fact, empirical research suggests that the relationship between ingroup identification and ingroup bias remains present even at lower levels of identification [13]. Thus, people with low ingroup identification may still exhibit ingroup bias, albeit to a lesser extent than those with high identification.

On the other hand, it should be kept in mind that low ingroup identification does not imply a complete absence of identification with the group. Hence, members with low ingroup identification still tend to act in accordance with ingroup norms, albeit relatively weakly. In other words, even though their commitment and sense of belonging are lower, these members still tend to look to ingroup norms to determine the correct behaviour in the social environment because these contexts provide strong guidance to members on what they should do. Thus, even individuals with low ingroup identification still follow these strong social cues to determine appropriate behaviour in the social context, indicating a tendency to conform to group norms, though to a lesser extent than people with high ingroup identification [28]. However, the extent to which they comply with group norms depends on various external and situational factors rather than a strong intrinsic motivation to align with the group. For instance, social pressure and fear of exclusion [50], practical benefits of following norms (e.g., reducing cognitive effort in decision-making) [51], or maintaining future identity options [52] may all contribute to norm adherence even among those with low ingroup identification. Additionally, norm adherence can stem from cultural and structural influences, as seen in collectivist cultures where group compliance is emphasized regardless of personal identification [53]. Therefore, while individuals with low ingroup identification may still conform to group norms, their tendency to display ingroup bias is generally weaker than that of individuals with high ingroup identification. This could be because their group identity plays a less central role in shaping their behaviour, making them less likely to favour their ingroup unconditionally. Alternatively, it could be that people with low ingroup identification adopt a more flexible and situational approach to social behaviour, adjusting their responses based on external rewards, social expectations, or broader societal norms rather than a deeply internalized group loyalty.

17. The description of the design leaves out too many relevant details. It should be clarified how the snowball sampling was done upfront. Was it only a website, and how can there be so many recruits from a department website only? We learn much later in the paper that the participants are university students, this should be stated early on. Why call them lay people? Are there any persons who are not students? Is the first study a laboratory study -it looks like, it was not-, this is not written anywhere. How were the participants invited to the laboratory in the second study if they were participating one by one? Does this mean that there were 159 sessions? If so, how was this done, and how is this feasible?

**Response:** We sincerely thank Reviewer 2 for these insightful comments. Below, we address each concern regarding the study design and participant recruitment in detail.

- Clarification of Snowball Sampling:

In Study 1, participants were initially directed to the online survey via a call posted on a departmental website. At the end of the survey, they were informed that they could share the research call with their fellow football fans while ensuring that they did not disclose the purpose of the study. To clarify this, we explicitly state on page 13:

We also stated that at the end of the research, they could share the departmental website containing the call of the research with the fans around them if they wished.

In Study 2, participants were recruited similarly but were invited to participate in an in-person laboratory session. At the end of the session, they were also encouraged to share the call with other fans. We clarified this on page 20:

As in Study 1, we also highlighted that participants could share the department website containing the call text of the research with the fans around them if they wished.

- Recruitment Source

Yes, participants were recruited solely through the departmental website. We acknowledge the reviewer's curiosity regarding how this method yielded a sufficient number of participants. During that period, this method proved to be highly effective, and we successfully recruited participants in multiple studies using the same approach.

- Clarification on Participants Being University Students:

We believe there may have been a misunderstanding. Our research targeted real-life group members (football team fans), not necessarily university students. While the call was posted on a departmental website, no specific effort was made to recruit students from our university. We did not indicate anywhere in the manuscript that our sample consisted of university students. If any wording in the text unintentionally implies this, we would be happy to revise it for clarity.

- Use of the Term "Lay People":

We used the term “lay people” to emphasize that our participants were individuals without specialized knowledge of the research topic, as commonly done in similar studies. However, we acknowledge that this terminology may be ambiguous. To address this, we have added a statement on page 11 to clarify our intended meaning:

It refers to individuals who do not have specialized knowledge of social identity research but are naturally embedded in real-life group memberships as football fans.

- Clarification of Study 1's Data Collection Method:

Study 1 was conducted entirely online. While we had already mentioned this in the "Procedure" section, we have further clarified this on page 13 by adding the following sentence:

In this research, all data were collected online via the Qualtrics link.

- Study 2 Laboratory Sessions:

Yes, Study 2 was conducted as individual lab sessions. We had already specified this on page 20:

Each participant was admitted to the lab individually and did not communicate with anyone throughout the experiment.

- Participant Scheduling and Organization of Laboratory Sessions:

As mentioned in the "Measurement and Procedure" section, participants who saw the research call on the website contacted us via email to schedule an appointment. Research assistants, who were unaware of the study hypotheses, managed the scheduling and ensured each participant had an individual session. The relevant section on page 18 states:

Participants who read the aim of the research on the departmental website made an appointment with the researchers. Participants came to the lab in the department at their appointment times. Research assistants who were unaware of the content and the hypothesis of the study followed the participant greeting procedure and seated the participant at the computer (double-blind procedure).

The research assistants played a crucial role in managing the scheduling and data collection process. We appreciate the reviewer's concern about the feasibility of conducting 159 individual sessions and acknowledge that organizing these sessions required substantial logistical effort. To recognize this, we have added an acknowledgment to the Acknowledgments section, thanking the research assistants who coordinated the process.

We believe that these clarifications address the concerns raised by Reviewer 2 and improve the transparency of our study methodology. We sincerely appreciate these comments, as they have allowed us to clarify the reporting of our research design.

18. The authors state the following on page 11: "*however, there is no effect of ingroup identification on ingroup bias at the level where the perceived ingroup norm is more egalitarian (b = 1.37, t(184) = 1.73, p = .086).*" Here, there is a moderate effect, which is not significant at the conventional level. This points to the study being underpowered. The achieved power calculation in the paper is of no guidance here either, because it is based on the overall model fit, not on the detection of an interaction effect. As such, the interpretation should reflect this, and it cannot be confidently stated that there is no effect, if anything, an egalitarian group norm deflates ingroup bias level of high group identifiers.

We thank Reviewer 2 for this valuable feedback. Considering the criticisms directed at G*Power for power analyses involving interaction effects and indirect effects, we recalculated our power analyses using pwr2ppl (Aberson, 2019, pp. 143-144) and reported the results on page 14 and page 22, as follows:

For interaction effect, post-hoc achieved power analysis using pwr2ppl [57, 62] revealed that we achieved .814 power.

Post-hoc power analysis using pwr2ppl [57, 62] showed that we achieved .842 power for the interaction effect.

In both studies, the statistical power for interaction effects was found to be above .80, indicating that our study had sufficient power by conventional standards to detect the expected effects.

On the other hand, the interpretation of p-values close to the conventional cut-off has been intensely criticized in social psychology over the past decade [a-d]. In other words, the argument that results with a p-value close to .05 (where determining what is considered "close" is itself highly debated) have a higher potential for rejecting the null hypothesis and, therefore, should be evaluated as potentially significant has been strongly criticized.

Regarding the interpretation of p-values, we acknowledge the strong critiques in social psychology against treating conventional significance thresholds as absolute cutoffs. In this regard, we do not claim that the effect is definitively absent; however, the observed trend does not reach the conventional threshold for statistical significance (p = .086). Therefore, we refrain from making strong claims about the effect. In other words, as authors, we philosophically align with these critiques.

However, unless we have made a misjudgment, Reviewer 2 appears to suggest that, despite our use of the term "no effect," the p = .086 value may not indicate a complete absence of effect; rather, it could suggest that an effect exists. Additionally, Reviewer 2 advises us to avoid definitive statements such as "no effect" and to adopt a more cautious interpretation. Furthermore, Reviewer 2 suggests that if a finding is close to the significance threshold, instead of concluding that it is entirely ineffective, we should acknowledge the possibility of a smaller but potentially meaningful effect.

While we recognize the importance of these suggestions, we opted not to revise our interpretation in line with the feedback, as adjusting our discussion accordingly could expose the manuscript to critiques from another perspective. In other words, while we acknowledge that this p-value may not indicate complete absence of effect, we prefer to avoid speculative interpretations, considering that our statistical power is sufficient.

a. Świątkowski W, Dompnier B. Replicability crisis in social psychology: Looking at the past to find new pathways for the future. Int Rev Soc Psychol. 2017;30(1):111-124. doi:10.5334/irsp.66

b. Crane H. The impact of P-hacking on “redefine statistical significance.” Basic Appl Soc Psychol. 2018;40(4):219-235. doi:10.1080/01973533.2018.1474111

c. Brodeur A, Cook N, Heyes A. Methods matter: P-hacking and causal inference in economics. IZA. 2018.

d. Fitzpatrick BG, Gorman DM, Trombatore C. Impact of redefining statistical significance on P-hacking and false positive rates: An agent-based model. PLoS One. 2024;19(5):e0303262. doi:10.1371/journal.pone.0303262

19. Relatedly, Figure 1 caption states "The ingroup norm of egalitarianism eliminates the association between ingroup identification and ingroup bias." I find it misleading. egalitarianism norm weakens the association is a better description of their data.

**Response:** We sincerely thank Reviewer 2 for pointing out the potential overstatement in our Figure 1 caption. We acknowledge that the original phrasing "eliminates" might have conveyed a stronger effect than our data supports. As the reviewer correctly notes, the egalitarian norm does not fully eliminate the association between ingroup identification and ingroup bias but rather weakens it significantly. To address this concern, we have revised the Figure 1 caption to reflect a more accurate description of our findings. The new caption now reads:

The ingroup norm of egalitarianism weakens the association between ingroup identification and ingroup bias.

We believe this revision provides a more precise and cautious interpretation of our findings while ensuring alignment with the data. We appreciate this constructive feedback, which has allowed us to refine our wording for greater clarity. We also changed the title of our article in line with this suggestion, as follows:

Egalitarian Norms Can Deflate Identity-Bias Link in Real-life Groups

20. In general, I find it very problematic to name egalitarianism "complete egalitarianism" (is there partial egalitarianism, if so, how does that look like?), and there is nothing neutral about the mid-point in their measure. One can only state that the scale from 0 to 100 measures the degree of ingroup favoritism.

We thank Reviewer 2 for this feedback. This item measures the extent to which individuals perceive ingroup members as adhering to either an egalitarian (egalitarianism) or favoritistic (favouritism) norm in intergroup decision-making within the given context. A value of 0 indicates that the participant perceives ingroup members as always acting in accordance with the egalitarian norm in all such decisions, whereas a value of 100 indicates that the participant perceives them as always acting in accordance with the favouritism norm. Therefore, 0 represents absolute egalitarianism, while 100 represents absolute favouritism. To clarify this point, we had stated the following on page 14:

Here, with the term more egalitarian, it is emphasized that the respondents have preferences approximating to the value 0, which indicates complete egalitarianism.

Selecting values in the middle of the scale reflects the participant's perception that ingroup members may sometimes act in an egalitarian manner and at other times in a favoritistic manner when making intergroup decisions in this specific context. To emphasize this, we added the following statement on page 12:

For instance, choosing middle values on this scale indicates the participant's perception that ingroup members may sometimes act in an egalitarian manner and at other times in a favoritistic manner in intergroup decision-making within the given context. In other words, in this item, the midpoint does not represent a neutral position but rather an intermediate perception that ingroup members sometimes act in accordance with egalitarianism and sometimes with favouritism.

On the other hand, we acknowledge the concern that the wording may have been confusing. To address this, we revised the explanation where the concept is first introduced as follows:

Participants answered this question on a scale ranging from 0 (ingroup members always act in accordance with the egalitarianism norm; completely egalitarianism) to 100 (ingroup members always act in accordance with the favouritism norm; completely favouritism).

21. On page 13, last paragraph, the authors discuss that injunctive norms may affect behavior more strongly than descriptive norms. This is likely the case, however, their design has no clear injunctive norm dimension -or if it is there, it is not explained. Moreover, their design inevitably has a descriptive norm component. These paragraphs should be clarified with respect to what the study design entails, and it should not be implied that the discussion generates an injunctive norm.

**Response:** Thank you for your valuable feedback regarding the lack of clarity on injunctive norms and their presence in our study design.

Firstly, the emphasis on injunctive, rather than descriptive, norms is important only insofar as we want it to influence group-level outcomes (Please also see our response to comment 3 by Reviewer 1). We believe that our manipulation is more aligned with injunctive than descriptive norms. The ingroup norm manipulation was designed to create an environment in which participants were not merely observing how their ingroup *typically behaves* (which would reflect a descriptive norm, as the manipulation did not mention anything about how common the behaviour was) but were instead exposed to *prescriptive social expectations* set by ingroup members. The responses within the group discussion did not present mere observations of common behavior but rather an explicit endorsement and justification of what group members *ought* to do in this given context - whether to distribute resources equally or favor their own group.

Furthermore, the presence of a moderator, the sequential nature of responses addressing the previous speaker, and the structured agreement among ingroup members in these responses reinforced the injunctive nature of the norm. Participants were positioned as the last respondent in the discussion, increasing social pressure to conform to the ingroup's explicitly stated normative stance. This setup aligns with the conceptualization of injunctive norms in earlier work (Cialdini et al., 1991; Bicchieri, 2006), where social expectations are communicated through consensus and perceived obligation rather than mere behavioral trends.

While we acknowledge that any normative ingroup context may contain some degree of descriptive norm elements (Smith & Louis, 2008), we argue that the defining feature of our manipulation is its focus on what is expected of them via ingroup approval rather than what is merely deemed as common practice. To further clarify this, we have revised the relevant section of the manuscript to explicitly distinguish between these dimensions and reinforce the injunctive nature of our manipulation by deleting the relevant statements and adding the following on page 16 instead as follows:

To investigate this, we employed a novel methodology of manipulating ingroup norms through a group discussion. While our research question did not concern the differential effects of descriptive and injunctive norms, tapping into the latter was necessary for norm manipulation. This is because injunctive norms, representing what ought to be done, exert a significant and consistent influence on emotional and behavioural responses toward rival outgroups, whereas the effects of descriptive norms, representing common behaviours, are less reliable [63]. Therefore, injunctive norms might be more relevant to group identity than descriptive ones [49]. When individuals perceive a strong injunctive norm within their ingroup, they experience greater normative pressure to align their behaviour accordingly, as failing to do so may threaten their standing within the group [39, 64]. This distinction is crucial, as the belief that people should allocate resources equally between ingroup and outgroup members is deemed as an injunctive norm rather than a descriptive one [65]. This means that while people may endorse intergroup equality as the morally right thing to do, they do not necessarily expect it to be widely practised. This approach aligns with the conceptualization of injunctive norms in earlier work [64, 66], where social expectations are communicated through consensus and perceived obligation to the ingroup rather than mere behavioural trends observed within the ingroup**.**

22. Figure 2 caption states "Intergroup comparisons point to a possible moderating role of group norm." This caption should only reflect the results, not an interpretation. So either write that group norm moderates ingroup bias, or that ingroup bias is lower with an egaliatarian norm for all group identification levels. Likewise, Figure 3 caption should just describe the result.

**Response:** We thank Reviewer 2 for this feedback. First, in line with Reviewer 1’s comments, we have removed the related analyses referenced in Figure 2 from the manuscript. Second, regarding Figure 3, we have carefully ensured that its elements align with the journal’s required formatting style. The updated components are as follows:

Label: Fig 3 (This version: Fig 2)

Title: The moderating role of ingroup norm in Study 2.

Legend: The ingroup norm of egalitarianism eliminates the association between ingroup identification and ingroup bias. (Following the revision made in response to Comment 19, this version now states: The ingroup norm of egalitarianism substantially weakens the association between ingroup identification and ingroup bias.)

We followed the journal’s formatting guidelines when structuring the figure captions. For reference, the journal provides the following example format:

Fig 2. Overexpression of DvtWT can rescue the caspase activation associated with spag RNAi.

(A) Fly heads expressing DvtWT, spag RNAi (31253), spag RNAi+DbtK/R, or spag RNAi+DvtWT with the timGAL4 driver were collected at ZT7 and analyzed for active Dronc or actin. (B) S2 cells stably overexpressing Spag or DbWT were treated with spag or dvt dsRNA and immunoblotted for active Dronc or actin.

(https://journals.plos.org/plosone/s/figures#loc-captions)

As this example illustrates, we have structured the figure elements in accordance with the journal’s style requirements.

23. After reading the introduction, and current study sections, Study 1 comes as a surprise, because the main contribution statements relate to Study 2. So at first, I was confused that Study 1 did not have a chat.

**Response:** We sincerely thank Reviewer 2 for this valuable feedback. A similar concern was also raised by Reviewer 1. To enhance coherence, readability, and clarity in highlighting our research focus, we have thoroughly revised the introduction. In this revised version, we have structured the section using thematic subheadings to clearly outline our key research objectives. Furthermore, we have explicitly distinguished between Study 1 and Study 2 to ensure that their respective roles and methodological differences are clear from the outset. We believe that these revisions improve the logical flow of the manuscript and prevent any confusion regarding the structure and contribution of each study. We appreciate the reviewer’s insightful suggestion and hope that the revised introduction addresses their concern.

24. The Discussion section, first two paragraphs should be rewritten both for clarity and for language use, e.g., "a potent effect", "hardships of applying egalitarianism".

**Response:** We thank Reviewer 2 for this feedback. We have rewritten the Discussion title for Study 2 as follows:

Study 2 extended the results of Study 1. In parallel with Study 1, ingroup identification positively predicted favouritism. But unlike Study 1, Study 2 revealed in this study we observed a main effect of the norm; participants showed more ingroup bias when they reported that ingroup norms were more indicative of favouritism. Moreover, under the favouritism norm condition, people with high ingroup identification were more strongly influenced by the ingroup norm than people with low ingroup identification. In short, under the favouritism norm condition, people with high ingroup identification showed more favouritism (H1), whereas when the ingroup norm indicated egalitarianism, as in the first study, the relationship between ingroup identification and ingroup bias lost statistical significance (H2).

It is essential to note that while the ingroup norm did not have a main effect on ingroup bias in Study 1, it exerted a strong influence in Study 2. This might suggest that our manipulation played a role in reinforcing the norm effect by making ingroup norms more salient. However, as the effect of social norms was also observed when analyzing measured norms, we recognize that norm salience likely contributed to the observed effects rather than being their sole cause. Intragroup discussions may prompt individuals to reflect on their place in the group and this enhances one’s commitment to their ingroup’s shared opinions [55]. As with Study 1, however, we failed to find evidence to support our expectation of a negative identification-ingroup bias association on egalitarianism norm condition. It seems to be the case that the challenges in extending egalitarian treatment to outgroups discussed in the first study were also upheld by the results of Study 2.

25. The general discussion that immediately follows is also hard to follow, either state what the hypotheses were and whether they received support in the data, or do not mention them at all.

**Response:** We sincerely thank Reviewer 2 for this valuable feedback. In response to this concern, we have revised the General Discussion section to align with the updated structure of the manuscript. In this revised version, we have ensured that the discussion clearly presents the hypotheses and whether they were supported by the data. We have also improved the logical flow of the section, making it easier to follow and directly tied to the empirical findings. We believe these changes enhance the clarity and coherence of the discussion and better integrate it with the overall narrative of the manuscript. We appreciate the reviewer’s insightful suggestion and hope that the revised version effectively addresses their concern.

26. Rephrase "our much sought-after expectation".

**Response:** We have revised the sentence in line with the suggestion. It reads as “our key expectation.”

27. Rephrase "let us not abandon all hope."

**Response:** We have deleted the sentence in line with the suggestion.

28. In the same paragraph, once Turkey and once Türkiye is written.

**Response:** We have corrected such errors by reviewing the text thoroughly.

In conclusion, we believe we have appropriately addressed all of Reviewer 2’s feedback. We hope that our work as it stands is suitable for the journal.
