# Supplementary material for: Egalitarian norms can deflate identity-bias link in real-life groups
Source: PLoS One. 2025 Aug 28;20(8):e0330484. doi: 10.1371/journal.pone.0330484 (PMC12393728; doi:10.1371/journal.pone.0330484)
Supplement: Attachment — Submitted filename: Review Letter - Round 3 V2.docx [file pone.0330484.s003.docx]

**Response Letter**

Dear Editor,

We are grateful to reviewers’ constructive feedback on our paper. To address reviewers’ concerns, first, we reorganized and shorten introduction and general discussion. Second, we have added the ANOVA results to Study 2 and re-checked the General Discussion heading to ensure that it aligns with all of these findings. Below, we have re-numbered each comments of Reviewers and provided our answers. We hope that this version of the paper is suitable for the journal.

Reviewer #1

The authors have made considerable efforts to improve the manuscript by taking into account all the suggestions provided by the reviewers. Furthermore, as stated in my previous review, I think that this research deserves to be published – possibly in a high impact journal such as Plos One. Besides these premises, unfortunately the current version of the article is still not fully clear, some sections and arguments are not fully convincing, and, most importantly, there is still ambiguity in the reported results.

1. In Study 2, the authors have deleted 2 X 2 ANOVA results, and rather just kept findings with measured identification and norms. I am aware that I made a comment about inconsistency in reporting findings which has probably been misinterpreted and which has led to this choice, and I am sorry about it. However, in my view this choice makes no sense. Study 2 is an experiment, and main data analyses need to focus on effects of experimentally manipulated variables, i.e. identification and norms. Otherwise, why conducting an experiment? I would strongly recommend that the authors report findings of the effects of experimentally manipulated norms, identification, and their interaction. Please note that the discussion includes several sentences about the effects of experimentally manipulated variables (likely kept from the previous version of the discussion) which make no sense as such findings are not reported.

**Response:** We sincerely thank Reviewer 1 for this important and constructive comment. Upon reflection, we realize that we had misunderstood their previous remark regarding inconsistencies in reporting findings, which led us to remove the 2 x 2 ANOVA from Study 2 in the revised version. We apologize for this misinterpretation and for the unintended consequence of weakening the alignment between our experimental design and the statistical analyses reported. As they rightly noted, Study 2 employed an experimental design in which both ingroup identification and ingroup norm were manipulated. We completely agree that the primary analyses in such a design should focus on the effects of these experimentally manipulated variables. In response to your current feedback, we have now re-included the full 2 x 2 between-subjects ANOVA in the Results section of Study 2.

This analysis tested the main and interaction effects of manipulated ingroup identification (high vs. low) and ingroup norm (favouritism vs. egalitarianism) on ingroup bias. The results showed significant main effects for both manipulated ingroup identification and manipulated ingroup norm, indicating that participants with high ingroup identification exhibited more ingroup bias than those in the low identification condition, and participants under the favouritism norm condition showed more ingroup bias than those under the egalitarianism norm condition.

Although the interaction effect was not statistically significant, we conducted pre-planned post-hoc comparisons to further explore the potential moderating role of identification, as hypothesized. At this point, we would like to emphasize one issue. We are familiar with the fact that post-hoc comparisons are generally not performed if an interaction effect is not statistically significant, but we do not disregard the recommendations in the literature that post-hoc comparisons should be conducted even if they are not statistically significant, especially if there is an expected interaction or if it is considered theoretically important. In our study, the statement that post-hoc comparisons were performed to identify a possible moderating role clearly indicates that there is a theoretical justification for this approach. In other words, as expected in the literature (see Çoksan & Cingöz Ulu, 2022; Jetten et al., 1997), we performed post-hoc comparisons for the interaction, even if it was not statistically significant, in order to further examine a possible moderating role. We would like to emphasize this justification again here. In conclusion, these comparisons revealed that in both high and low identification conditions, participants exposed to the favouritism norm showed significantly more ingroup bias than those exposed to the egalitarian norm. Moreover, under the favouritism norm condition, participants with high ingroup identification displayed more ingroup bias than those with low identification, whereas no significant difference was observed under the egalitarian norm condition.

In light of these findings, we have also thoroughly revised the General Discussion heading. Specifically, we removed or rephrased any statements that referenced experimental effects without supporting results in the previous version. The current version now accurately reflects the re-inserted statistical findings and draws conclusions that are fully grounded in the reported analyses. We also wish to highlight that the decision to conduct these post-hoc comparisons was not data-driven but theoretically justified and planned in advance, as our hypotheses involved the moderation of identification by group norms. We have clarified this rationale in the Methods and Results sections as well.

We are grateful for Reviewer 1’s feedback, which significantly improved the methodological integrity and reporting transparency of our manuscript. Their input helped us ensure that our statistical reporting is appropriately aligned with our experimental design and theoretical aims. If any additional clarifications or improvements are needed, we would be happy to address them as well.

2a. The argument that the experimental manipulation of social norms in Study 2 taps injunctive norms more than descriptive norms is not convincing. Participants read the opinion of other ingroup members (football fans supporting the same team) about how advertising revenues should be distributed, but I guess that in the real world such distribution is determined by the league and by negotiations between the league and the football clubs, with very limited voice by football fans. Therefore, the norms experimental manipulation is not much about how ingroup members (football fans supporting the same team) should act but rather about what ingroup members think. In my view this aligns more with descriptive rather than injunctive norms.

**Response:** We appreciate the reviewer’s attention to the distinction between injunctive and descriptive norms, and we think that they often overlap in real-life settings. We do not claim that our manipulation taps *purely* or *exclusively* into injunctive norms. In fact, we acknowledge that elements of both descriptive and injunctive norm communication are embedded in our design; as is often the case when group members express their views about a behavior’s typicality and the necessity to endorse it. For instance, we asked participants whether they believed that “most of the people I spoke to *think* that we *should* treat all parties equally in the allocation of resources such as advertising revenues,” capturing both perceived consensus (a descriptive element) and prescriptive belief (an injunctive element).

That said, we maintain that our manipulation aligns more closely with injunctive norms for 4 reasons:

a. In both egalitarianism and favouritism conditions, the chat structure explicitly frames the conversation around what ingroup members *should* do. The moderator’s questions repeatedly ask for judgments of what the Turkish Football Federation ought to do with revenue allocation, and more importantly, whether participants believe that *supporters of their team* value that approach. This framing encourages reflection on collective moral obligation, not just observed behavior.

In participants’ responses, this moralizing obligation on the group is evident. In the egalitarian condition, for example, participants say that “giving more share to a team does not befit our fans” and “equal shares means equal fighting power.” Similarly, in the favouritism condition, participants state that “you’re hurting your own team” if you don't advocate for more funding to your team, and that “In terms of fair play, it is important that such a resource is transferred more to the teams that create that resource.” These are not mere reports of common practice but expressions of what is appropriate or expected from group members; inducing a sense that it is the right thing to do for the ingroup.

b. We respectfully disagree with the characterization of the manipulation as participants “reading the opinion of other ingroup members.” This interpretation overlooks critical aspects of the procedure that distinguish it from a passive exposure paradigm (e.g., reading a forum or article). In our design, participants took part in a time-paced, simulated group chat moderated by a researcher. They were called to engage with the conversation three times by contributing their own views after seeing how other ingroup members had already articulated a stance. This setting mimics an interactive group discussion, emphasizing active participation rather than observational learning.

The chat structure itself promotes consensus-building. Participants responded in a fixed order, allowing the first speaker to establish a normative tone that subsequent speakers then addressed and affirmed and elaborated upon, instead of speaking up their mind with no attention to the previous speaker. In the egalitarian condition, for instance, the second speaker not only showed agreement with the first speaker's response but provided additional justifications rooted in group values (e.g., fairness, equality, collective responsibility). A parallel structure was observed in the favouritism condition, where speakers reinforced the moral importance of supporting their own team. This structure reflects how injunctive norms are formed and maintained: through group discussions that converge on shared expectations via moralizing group reasoning.

c. We agree with the reviewer that football fans have little to no formal control over the actual distribution of money between football clubs. However, this lack of direct influence does not undermine the function of injunctive norms. Actions, even those that appear irrational from a purely instrumental perspective (e.g., minimal group paradigm), can serve to affirm group membership and group’s positive distinctiveness.

Normative influence, especially injunctive norms, does not depend on individuals’ ability to directly affect policy or outcomes. Even when people cannot materially change a situation, they often express what they believe their group *should* do (Jaeger & Schultz, 2017). In our study, participants routinely refer to group-based ideals (e.g., “this is what our fans value,” “it’s important for fair play”), indicating an orientation toward group-based moral consensus rather than strategic efficacy. The normative expectations they voiced were less about influencing real-world policy and more about signaling identity-based commitments and obligations within their ingroup. In fact, as previous research has shown, injunctive group norms can emerge precisely in contexts where individuals rely on group-based moral discourse rather than perceived efficacy to influence outcomes (e.g., Acar et al., 2024; Workman et al., 2020). This indicates that normative influence can stem from internalized group norms rather than the ability to directly influence policy.

Thus, while fans may lack direct power over league decisions, their alignment with injunctive norms remains meaningful and, we argue, central to how the manipulation operates.

d. The content of the conversations in our experiment revolves around fairness, deservingness, and group values, all of which are central to injunctive norm perception (Heuver et al., 1999; Juan-Bartroli, 2024). Crucially, this emphasis holds regardless of the direction of the normative stance. Whether participants advocate for equal distribution or favoring their own team, their justifications are rooted in what they believe to be right, deserved, or morally appropriate, more than in what is merely common or typical.

In sum, while the manipulation incorporates both norm elements, we believe it is more aligned with injunctive norms due to its structure and content. Considering the request to further shorten the manuscript, we have opted not to include these additional explanations in the main text.

In conclusion, while the chat content reflects the perceived views of ingroup members, the structure of the questions and participants’ own responses go beyond mere observation of others’ opinions. The wording used and the moral reasoning in their responses indicate not just perceived typical behavior, but shared group obligations. Therefore, the manipulation invites participants to deliberate on what is appropriate and expected, not merely on what is common. For instance, the contents that are used during the ingroup norm manipulation task were as follows:

MODERATOR DENIZ:

Hello, I’m Deniz, the researcher, and I’ll be moderating this discussion. My request is that you answer only the questions I ask, in the specified order. For each question, please respond in this sequence: 1) Kraker1, 2) sonütücü, 3) Kalemşör, 4) ${q://QID91/ChoiceTextEntryValue}. You may need to scroll down to see what I’m about to write, as it may appear at the bottom of your browser.

MODERATOR DENIZ:

I’ll now ask you some questions about how you think the Turkish Football Federation (TFF) should distribute its advertising revenue. But first, I’d like to provide some background information on TFF’s revenue streams.

Once everyone is ready, you can click the "Next" button that will appear at the bottom right.

MODERATOR DENIZ:

The Turkish Football Federation is the sole authorized organization to sell broadcasting rights for matches of teams competing in the Turkish Süper Lig to TV channels, radio stations, and social media platforms. The federation generates income by selling these broadcasting rights. Additionally, they receive a share of the revenue from product advertisements shown to consumers during matches. With this substantial income, TFF distributes a portion of the revenue among the league’s teams.

MODERATOR DENIZ:

This distribution process sometimes sparks debate. Some fans argue that all teams should receive equal shares, while others believe more investment should go to financially disadvantaged teams to foster football development. On the other hand, some fans claim that teams with higher viewership should receive larger shares since their matches attract more attention. This revenue is one of the income sources for the league’s teams.

MODERATOR DENIZ:

My first question is: How do you think TFF should distribute this advertising revenue? You may answer in order.

Kraker1:

I believe our team’s fans value equality in this matter. Whether it’s a big club or an Anatolian team, it shouldn’t matter. This revenue comes from all teams, so it should be distributed equally.

sonütücü:

I think similarly. Yes, some teams get more coverage in newspapers and on TV, but private companies already direct their ads to those teams. Other revenue exists because there’s a league. If the league is a whole and the teams are its stakeholders, at least this income should be shared equally.

Kalemşör:

I also support equal distribution. Why should one team get a larger share? Even if some teams appear more in ads, those ads aren’t only watched by their fans—the revenue comes from all viewers. Besides, favoring one team, whether ours or not, wouldn’t sit right with our fanbase.

${q://QID91/ChoiceTextEntryValue} IT’S YOUR TURN. AFTER WRITING YOUR ANSWER IN THE BOX BELOW, YOU CAN CLICK THE "NEXT" BUTTON THAT WILL APPEAR AT THE BOTTOM RIGHT.

MODERATOR DENIZ:

Thank you for your responses. Do you think equal distribution of this revenue is important for your team’s fans? What are your thoughts? You may answer in order.

Kraker1:

I think it’s important. Otherwise, it’d be favoritism. Equal shares mean equal competitive power; the better team wins. This boosts people’s trust in sports.

sonütücü:

I agree. Equal distribution matters for fair play. Fans like us value this fairness.

Kalemşör:

I believe we prioritize equality. Sure, some fans might prioritize their team’s interests, but they’re a minority. Most of us would support equal shares.

${q://QID91/ChoiceTextEntryValue} IT’S YOUR TURN. AFTER WRITING YOUR ANSWER IN THE BOX BELOW, YOU CAN CLICK THE "NEXT" BUTTON THAT WILL APPEAR AT THE BOTTOM RIGHT.

MODERATOR DENIZ:

Do your opinions apply to all of TFF’s advertising revenue sources (social media, TV, etc.)? You may answer in order.

Kraker1:

Yes, they do. Equality shouldn’t depend on the source. As I said earlier, the shares should be equal.

sonütücü:

The source doesn’t matter. There’s a collective revenue pool contributed to by all teams’ fans. Why not distribute it equally? Some teams have more fans, but giving them more resources would disrupt fairness, widen financial gaps, and weaken smaller teams’ competitiveness. For fair play, the source shouldn’t matter—every team should get an equal share.

Kalemşör:

I generally agree. The source isn’t important. Equality is key for a fair league.

${q://QID91/ChoiceTextEntryValue} IT’S YOUR TURN. AFTER WRITING YOUR ANSWER IN THE BOX BELOW, YOU CAN CLICK THE "NEXT" BUTTON THAT WILL APPEAR AT THE BOTTOM RIGHT.

MODERATOR DENIZ:

Thank you for your responses. Our written discussion ends here. Next, we’d like you to answer some questions on your computer screen regarding TFF’s revenue distribution policies.

You can proceed by clicking the button that will appear at the bottom right.

As seen in the content, other fans respond by emphasizing *we,* indicating how a team’s supporters might collectively behave in a given situation, which we believe points to an ingroup norm. On the other hand, we understand Reviewer 1's concern and recognize that readers may share this worry. Therefore, we have added an example of norm manipulation to the article's OSF page. On page 21, we addressed this issue as follows:

As we stated above, all chat procedure (see Appendix I) was predetermined and automatically run through Java Script coding in the Qualtrics interface.

Additionally, we have included a brief explanation in the text about how these norms were defined, as follows:

Although our manipulation draws on both descriptive and injunctive aspects, it was framed and received by participants in a way that encouraged reflection on appropriate rather than typical behavior.

Moreover, to highlight that participants were not passive observers but were prompted three times to contribute their own views within a simulated group chat and their answers reflected elaborated reasoning aligned with what they believed should guide ingroup behavior; hence this setting enabled co-construction of norms, a process characteristic of injunctive norm formation, we added the following sentence:

Participants were explicitly asked to respond in the chat, after viewing others’ comments, which encouraged them to actively formulate their own position regarding what ingroup members should support.

Acar, Y., Figueiredo, A., Kutlaca, M., Li, M., Mustafa, A., Sagherian-Dickey, T., Vestergren, S., & Vollhardt, J. R. (2024). Why protest when it is not working? The complexities of efficacy in the current Palestine solidarity protests. *Social Psychological Review*, *26*(1), 10-17. <https://doi.org/10.53841/bpsspr.2024.26.1.10>

Heuer, L., Blumenthal, E., Douglas, A., & Weinblatt, T. (1999). A Deservingness approach to respect as a relationally based fairness judgment. *Personality and Social Psychology Bulletin*, *25*(10), 1279-1292. <https://doi.org/10.1177/0146167299258009>

Jaeger, C. M., & Schultz, P. W. (2017). Coupling social norms and commitments: Testing the underdetected nature of social influence. *Journal of Environmental Psychology*, *51*, 199-208. <https://doi.org/10.1016/j.jenvp.2017.03.015>

Juan-Bartroli, P. (2024). On injunctive norms: Theory and experiment. *TSE Working Paper*, n. 24-1515. <https://www.tse-fr.eu/sites/default/files/TSE/documents/doc/wp/2024/wp_tse_1515.pdf>

Workman, C. I., Yoder, K. J., & Decety, J. (2020). The dark side of morality – Neural mechanisms underpinning moral convictions and support for violence. *AJOB Neuroscience*, *11*(4), 269-284. <https://doi.org/10.1080/21507740.2020.1811798>

2b. Relatedly, the authors are still referring to the experimental manipulation as “interactive” repeatedly throughout the manuscript. There was no actual interaction, because participants just read the opinion of others and expressed their opinion. While I find the experimental manipulation through a simulated discussion very thoughtful and creative, it should not be described as “interactive”.

We sincerely thank Reviewer 1 for pointing this out. Upon reflection, we agree that referring to the manipulation as *interactive* might unintentionally suggest a real-time or two-way exchange among participants, which was not the case in our study. Although our intention was to convey that participants were engaging with pre-existing group discourse and were actively prompted to respond within a simulated group discussion environment, we acknowledge that the term *interactive* may be misleading. Therefore, we have revised the manuscript by replacing the term *interactive* with more accurate descriptor, which is *simulated group discussion*. These terms better reflect the nature of the task, in which participants read scripted comments from ostensible group members and were then required to contribute their own views in a structured, timed format. We appreciate Reviewer 1’s thoughtful and constructive feedback, which helped us improve the clarity and accuracy of our methodological description.

3. The argument that the intergroup context is not conflictual is still not fully convincing. I agree that the distribution of resources task used in the outcome variable is not conflictual, but relationships between fans of different football teams can be conflictual.

**Response:** We thank Reviewer 1 for raising this valuable point. We understand that the nature of the intergroup context in our study required further clarification. While we initially described the context as *non-conflictual,* we agree that intergroup relations between fans of different football teams can sometimes involve elements of conflict, especially in certain cultural or national contexts. To better reflect this nuance, we have revised our description to state that Turkish football fans *represent real-life groups engaged in competitive but not overtly conflictual intergroup relations.* This phrasing acknowledges the competitive nature of the group dynamics without overstating the absence of conflict. It also more accurately positions our work in relation to previous studies conducted in high-conflict or minimal-group contexts. We appreciate their observation, which prompted us to improve the precision and nuance of our description.

4. Regarding the organization of the introduction, I think that the authors have improved it a lot compared to the previous version. However, readability could be further improved. I would recommend that the authors clearly define the following concepts, before mentioning them for the first time: differentiation, favoritism, fairness, egalitarianism. While I agree in the distinction between fairness and egalitarianism, and that egalitarianism might be a stronger means to counteract intergroup bias than fairness, the rhetoric on the distinction between the two could be clearer and more straightforward to follow.

**Response:** We thank Reviewer 1 for their encouraging words about the improved structure of the introduction and for pointing out an important area for further clarification. In response to their feedback, we have now added brief and clear definitions of the concepts “differentiation,” “favoritism,” “fairness,” and “egalitarianism” at the point where each is introduced in the introduction. We also revised the paragraph where we contrast fairness and egalitarianism, aiming to clarify this distinction in a more direct and accessible manner. The updated version highlights that while fairness involves giving individuals what they deserve based on merit, egalitarianism emphasizes strict equality of outcomes, which may offer a more robust path to reducing intergroup bias. We appreciate this suggestion, which allowed us to improve the clarity and readability of the conceptual framework.

5. The general discussion needs to be thoroughly revised after the implementation of the other changes, especially point 1.

**Response:** We thank Reviewer 1 for drawing attention to the importance of aligning the General Discussion with the updated results, especially following the reintegration of the 2 x 2 ANOVA findings. In response, we carefully reviewed and revised the General Discussion section to ensure consistency between the reported results and the interpretations presented. Specifically, we rephrased or removed statements that no longer reflected the findings, particularly those referencing experimental effects that had not been included in the previous version. We also added new commentary grounded in the updated results, including the main effects of manipulated ingroup identification and ingroup norm, as well as the planned post-hoc comparisons. These revisions clarify the implications of our findings (especially in relation to the partial support and the lack of support for our hypotheses), and situate them within the broader literature. We hope the revised section now provides a more accurate and coherent synthesis of our theoretical contributions and empirical outcomes. We are grateful for encouraging us to strengthen this part of the manuscript.

6. Study 2 sample size might not be ideal given the 2 X 2 research design. It is also still unclear which power analysis was conducted.

**Response:** We thank Reviewer 1 for raising this important point. While we did not conduct an a priori power analysis for the 2 x 2 ANOVA before data collection, we have now performed a post-hoc power analysis based on the observed effect sizes in Study 2. Although we had already included detailed post-hoc power results for the moderation analyses, we agree that addressing power related to the experimental design is also important. The post-hoc analysis indicated that our sample size had high power for detecting the main effects, but relatively low power for detecting the interaction effect. We have acknowledged this limitation in the General Discussion section of the revised manuscript and provided further details in the Appendix II on the study’s OSF page. We hope this clarification addresses Reviewer 1’s concern.

7. When reporting decomposition of interactions, have the authors calculated simple effects at +1 and -1 standard deviation of the moderator? This needs to be clarified in the main text.

**Response:** We thank Reviewer 1 for pointing out the need to clarify our approach to decomposing interactions. In both Study 1 and Study 2, we conducted simple slopes analyses at one standard deviation above and below the mean of the moderator variable, following standard procedures (Aiken & West, 1991). However, we realize that this was not explicitly stated in the previous version of the manuscript. We have now clarified this point in the Results section to ensure transparency and interpretability of the moderation effects, as follows:

Simple slopes were calculated at one standard deviation above and below the mean of the moderator variable.

8. I do not agree with the interpretation of the mid-point of the social norms measure. Unless there was a label associated to the mid-point, it cannot be surely known that “the midpoint does not represent a neutral position but rather an intermediate perception that ingroup members sometimes act in accordance with egalitarianism and sometimes with favouritism”. It is also possible that the mid-point is interpreted as a neutral opinion. Please note that all the lines on the mid-point might not be necessary (see next comment).

**Response:** We thank Reviewer 1 for raising this important point regarding our interpretation of the midpoint of the social norms scale. We acknowledge that the midpoint was not labelled and may have been interpreted in different ways by participants, as a neutral position, an ambivalent view, or an absence of a strong opinion. In response to this feedback, we have revised the manuscript to reflect this ambiguity and have softened our interpretation. Specifically, we now clarify that midpoint responses could reflect either mixed perceptions about the norm or an uncertain stance, and we avoid drawing firm conclusions based solely on midpoint values. We hope this clarification addresses their concern.

9. While Plos One might have no word limit, I think that some sections could be shortened to make the article easier to read and follow. For example, in the introduction there is one page and a half on what happens to people with low identification, but such text sounds partly repetitive. Please note that the comment on the length of the paper is determined also by the fact that this research largely replicates the theoretical rationale, hypotheses and findings by Jetten et al. (1997) and by Çoksan and Cingöz-Ulu (2022). I believe that this research program deserves to be published, the article could be shorter and, most importantly, more straightforward.

**Response:** We thank Reviewer 1 for this thoughtful and constructive feedback. In response, we have thoroughly reviewed and revised the introduction heading to reduce redundancy and improve clarity. We also streamlined the language across the section to ensure that each paragraph adds distinct value to the conceptual framework without unnecessary repetition. Moreover, we agree that readability and conciseness are important; therefore, we took this opportunity to refine the manuscript to make it more straightforward and accessible. We hope these changes make the text more focused while retaining the theoretical richness necessary to frame our contribution.

10. Finally, there might still be some inaccuracies. For example, the sentence “the relationship between ingroup identification and ingroup bias remains present even at lower levels of identification” is imprecise. The whole article should be carefully re-checked after resubmission.

**Response:** We agree that the sentence *the relationship between ingroup identification and ingroup bias remains present even at lower levels of identification* lacked sufficient precision. We have revised the sentence to clarify that individuals with low levels of identification may still show ingroup bias, albeit to a lesser extent than high identifiers. Additionally, we have carefully reviewed the manuscript for any other vague or imprecise statements and have revised them to improve clarity and accuracy. We greatly appreciate Reviewer 1’s suggestion, which helped us enhance the conceptual and linguistic precision of the manuscript.

Reviewer #2: I would like to thank the authors for a much improved manuscript. I have only a few comments.

1. While the authors corrected the usage of the word "neutral" when referring to mid-value of their ingroup favoritism measure, the word stays in the figures. Please change the wording in the figures as well for a correct description.

**Response:** We thank Reviewer 2 for their careful attention to detail. As suggested, we have revised the figures in the manuscript to remove the term *neutral* from the midpoint labels. This change ensures consistency with the revised interpretation presented in the main text. The midpoint is now labeled numerically, without implying a neutral stance.

2. Please include in the manuscript, maybe in the Appendix, a few example discussions generated in Study 2. Any reader is left blind as to its contents.

**Response:** We thank Reviewer 2 for this helpful suggestion. We fully agree that providing illustrative examples from the group discussions used in Study 2 enhances transparency and allows readers to better evaluate the content and tone of the norm manipulations. This point was also implicitly raised by Reviewer 1, and in response to both Reviewers’ comments, we have now included the related example contents in the Appendix I of the revised manuscript on the OSF page of the paper. We believe this addition will provide a clearer understanding of how group norms were communicated in our experimental manipulation.

3. Describe in text what high and low refer to in the figures; are these the extremes, or grouped values? If the latter, what are the cutoffs?

**Response:** We thank Reviewer 2 for this helpful observation. In Study 1, the *high* and *low* levels of ingroup identification shown in Figure 1 represent values at +1 standard deviation and -1 standard deviation from the sample mean, respectively. We had previously noted this information in the text, but to enhance clarity, we have now explicitly labeled these values as *+1 SD*, *Mean*, and *-1 SD* directly on the figure. This should allow readers to more clearly interpret the decomposition of the interaction based on the continuous moderator. We appreciate the opportunity to improve the transparency of our visualizations.

4. "completely egalitarianism" should be "complete egalitarianism", and likewise, completely favouritism should be complete favouritism.

**Response:** We thank Reviewer 2 for pointing out the inappropriate phrasing. We have carefully reviewed the manuscript and replaced *completely egalitarianism* with *complete egalitarianism* and *completely favouritism* with *complete favouritism* to ensure grammatical accuracy. We appreciate their attention to detail.

5. In the first paragraph of the introduction, "In such resource allocation tasks, the perception of resource scarcity appears to promote the endorsement of zero-sum beliefs and, in turn, favouring the ingroup over outgroups [4].", I do not see how this is relevant to this work, especially in the opening paragraph. The resource allocation tasks are zero-sum, so referring to this finding generates a misleading idea of what the research is about.

**Response:** We thank Reviewer 2 for this insightful comment. We agree that the original phrasing in the introduction may have been misleading, especially considering that the resource allocation task in our study already operates under a zero-sum logic. In response, we have removed all references to zero-sum framing from both the introduction and the discussion sections of the manuscript. We believe this revision enhances conceptual clarity and ensures that the description of our task is more accurately aligned with the study’s actual design.

6. Change should to would or are expected to be in the following sentence, as you are making a prediction not a prescription for behavior: "These findings indicate that people with high ingroup identification should be more mindful of ingroup norms and accordingly revise their behaviour than people with low ingroup identification."

**Response:** We thank Reviewer 2 for pointing out the prescriptive tone in the sentence. As suggested, we revised the sentence to reflect the predictive nature of our claim. The revised version now reads: “These findings indicate that people with high ingroup identification are expected to be more mindful of ingroup norms and accordingly revise their behaviour than people with low ingroup identification.” This change improves the clarity of our intended meaning and aligns better with the language of scientific prediction.

7. "one of the few studies", not "one of the rare studies".

**Response:** We thank Reviewer 2 for this helpful linguistic suggestion. We have revised the phrasing from “one of the rare studies” to “one of the few studies” in the manuscript to ensure more natural and conventional academic wording.

8. Sentences with "we thought that" read unnatural and not particularly scientific. These words can be omitted.

**Response:** We appreciate Reviewer 2’s careful attention to the tone and style of our manuscript. In accordance with this suggestion, we have removed or rephrased all sentences that included “we thought that,” opting for more formal and scientific expressions to enhance the clarity and academic tone of the text.

9. I do not see how the study introduces cultural dynamics, and the following claim can be made: "In this way, we thought that we could both increase the generalizability of the findings and contribute to the understanding of cultural dynamics by filling this sampling gap in the literature."

**Response:** We thank Reviewer 2 for this insightful comment. We recognize that our previous wording may have overstated the cultural relevance of our sampling. To clarify, our intention was to highlight that by recruiting real-life group members from Türkiye, a context less frequently examined in this literature, we aimed to enhance the generalizability of findings beyond Western samples commonly used in studies on group norms and intergroup bias. However, we agree that this does not amount to a direct investigation of cultural dynamics. Accordingly, we have revised the sentence to more accurately reflect our contribution, stating that our study increases the cultural representativeness of the literature rather than directly examining cross-cultural processes.

10. I am not sure having different values mean in the following sentence, please specify or change wording: "Participants randomly assigned to a low ingroup identification condition, on the other hand, were informed that they have different values from their team’s other fans and were an atypical member of their ingroup."

**Response:** We thank Reviewer 2 for pointing out this ambiguity. Our original intention was to operationalize low ingroup identification by creating a sense of misalignment between the participant and the broader ingroup, thereby reducing perceived similarity and typicality. To address this concern, we have revised the sentence for clarity. The revised wording now reads: “Participants randomly assigned to the low ingroup identification condition were informed that, based on prior survey responses, their views and preferences differed from those of the majority of their team’s supporters, making them an atypical ingroup member.” We believe this revision more accurately conveys the manipulation without causing confusion about the meaning of “different values.”

11. Please complete this phrase after "to a lesser extent" indicating the comparison unit "Our main expectation that those with higher identification in the egalitarian norm would favour their ingroup to a lesser extent was not supported in either of the studies (H2)"

**Response**: We have revised the paragraph that covers our hypotheses.

12. How can the authors be sure that they did not draw ultra-fans to their study? The discussion makes such a claim.

**Response:** We thank Reviewer 2 for drawing attention to this important issue. We acknowledge that our sample consisted of self-identified football fans and that we cannot fully rule out variation in baseline levels of team commitment. While our experimental manipulation successfully created distinct levels of perceived ingroup identification, it remains possible that some participants were more intensely committed to their team than others. In response to this concern, we revised the relevant paragraph in the General Discussion to remove the reference to “ultra-fan groups” and to more cautiously acknowledge this limitation. The updated version now highlights the possibility of varying levels of baseline attachment and the potential implications this may have for the interpretation of our findings. We also note that in contexts involving stronger intergroup hostility, the role of ingroup norms may operate differently, which we suggest as an avenue for future research. We appreciate this suggestion, which helped us improve the nuance and accuracy of our discussion.
