# Supplementary material for: Egalitarian norms can deflate identity-bias link in real-life groups
Source: PLoS One. 2025 Aug 28;20(8):e0330484. doi: 10.1371/journal.pone.0330484 (PMC12393728; doi:10.1371/journal.pone.0330484)
Supplement: Attachment — Submitted filename: Review Letter - Round 4.docx [file pone.0330484.s004.docx]

**Response Letter**

Dear Editor,

We thank the Reviewer for their feedback. Below, we have numbered and responded to each of the six comments. We believe we have addressed all of the Reviewer’s concerns. To do so, we first provided a more detailed definition of the concept of fairness in the section where it is first introduced, ensuring consistency with the rest of the text. We revisited the points raised by the Reviewer regarding the reporting of results. In Study 2, we clearly stated our rationale for conducting a moderation analysis alongside the ANOVA to test our hypotheses. Finally, we carefully reviewed the manuscript sentence by sentence to ensure the language is flawless. We hope that the revised version is now suitable for publication.

Reviewer #1

The authors have thoroughly revised the article by taking into account the second round of comments by the reviewers. While the article is considerably improved, there are still some minor issues which need to be addressed.

1. The definition of fairness on page 3 is unclear and does not fully match what is stated later in the article about fairness.

**Response:** We thank Reviewer 1 for highlighting this issue. We compared the initial mention of the fairness norm on Page 3 with the section titled “Fairness vs. Egalitarianism” to ensure consistency in how the norm is defined. To make the first usage consistent with the more detailed explanation later in the manuscript, we briefly and clearly introduced the core elements included in that later definition, such as merit, proportionality, and need. This adjustment also helps clarify the flexible and context-dependent nature of the fairness norm. We believe that this revision ensures consistency throughout the manuscript. The revised statement on Page 3 now reads as follows:

The difference between people with high and low identification, however, disappeared when the fairness norm, which refers to collective standards within a group regarding what its members consider right, appropriate, or justified based on criteria such as merit, proportionality, or need, was in place. Fairness norms are thus context-dependent and may lead to unequal outcomes that are nonetheless perceived as legitimate or justified by group members.

2. Study 2 findings. The wording when reporting the decomposition is not fully clear. For example, the sentence “Post-hoc comparisons between experimental conditions indicated that participants with favoritism ingroup norm show more ingroup bias (N = 30, M = 5.21, SE = .93) than those with egalitarianism norm condition (N = 31, M = 2.15, SE = .92; p = .021, 95% CI [.476, 5.645]) on low ingroup identification condition.” Could be revised as something like “Post-hoc comparisons indicated that, among participants in the low ingroup identification condition, there was more ingroup bias among those in the favoritisms ingroup norm condition compared to those in the egalitarianism ingroup norm condition.” The following sentences should be revised as well for clarity and correctness.

**Response:** We thank Reviewer 1 for pointing out the need for greater clarity. We have revised the sentences in the Study 2 findings section as suggested. In this revised version, we simplified the sentence structure and clarified which groups were compared under which conditions. We emphasized the experimental ingroup norm conditions (favoritism norm, egalitarianism norm) and levels of identification (high identification, low identification). This allowed us to present the results more clearly and made the sentences easier to follow. These adjustments significantly enhance readability and ensure that our findings are accurately conveyed. The final version of the four sentences highlighted by Reviewer 1 appears on page 21 as follows:

Post-hoc comparisons indicated that, among participants in the low ingroup identification condition, there was more ingroup bias in the favoritism norm condition (*N* = 30, *M* = 5.21, *SE* = .93) compared to the egalitarianism norm condition (*N* = 31, *M* = 2.15, *SE* = .92; *p* = .021, 95% CI [.476, 5.645]). Similarly, among participants in the high ingroup identification condition, those in the favoritism norm condition (*N* = 32, *M* = 8.33, *SE* = .90) displayed greater ingroup bias compared to participants in the egalitarianism norm condition (*N* = 41, *M* = 2.86, *SE* = .80; *p* < .001, 95% CI [3.092, 7.852]). In the favoritism norm condition, participants with high ingroup identification (*M* = 8.33, *SE* = .90) showed more ingroup bias compared to those with low ingroup identification (*M* = 5.21, *SE* = .93; *p* = .017, 95% CI [.558, 5.686]). However, in the egalitarianism norm condition, there was no significant difference in ingroup bias between participants with high and low ingroup identification (*p* = .559, 95% CI [-1.690, 3.113]).

3. Still on Study 2 findings. As previously explained, the main moderation hypothesis needs to be tested with ANOVA and post-hoc comparisons. However, the authors have chosen to keep results reporting the interaction tested with cross-sectional data. If the authors want to keep that section, they need to clarify the goal of such analysis, and specifically how they justify a moderation test run with measures which were initially intended to act as manipulation checks.

**Response:** We thank Reviewer 1 for highlighting this important methodological issue. To clarify, the moderation analysis conducted with continuous scores on perceived ingroup identification and perceived ingroup norms (initially used as manipulation checks) was included to provide convergent validity and methodological triangulation of our experimental findings rather than independently testing our main hypotheses. Specifically, we now explicitly emphasize in the revised manuscript that this supplementary analysis:

(a) Serves primarily as an additional verification method to explore the robustness of our experimental results through an alternative analytic approach (see Hayes, 2022); (b) Allows us to examine how individual differences in participants’ subjective perceptions in response to manipulations relate to ingroup bias, thus enhancing our understanding of the moderation process; and (c) Provides methodological consistency and comparability between Study 1 (correlational) and Study 2 (experimental), enabling us to offer a more integrative narrative across both studies.

This clarification has been explicitly included in the Results section of Study 2, immediately following the ANOVA results and before presenting the supplementary moderation analysis. We greatly appreciate Reviewer 1’s insightful comment, as addressing it allowed us to enhance the methodological transparency and analytic coherence of our manuscript. The paragraph we added on Page 22 is as follows:

To provide convergent validity and methodological triangulation of our experimental findings, we additionally conducted a supplementary moderation analysis using participants’ continuous scores on the manipulation checks of perceived ingroup identification and perceived ingroup norms. It is important to clarify that although these measures initially served as manipulation checks, employing them in a moderation model allows us to examine the robustness of our experimental results through an alternative analytic strategy [65]. Specifically, while the ANOVA approach tested the causal impact of manipulated categorical variables, this complementary moderation analysis explores how individual differences in response to manipulations may be systematically related to ingroup bias. This supplementary analysis was preplanned and serves to support the primary experimental analyses rather than to independently test our hypotheses. Moreover, the moderation model conducted in Study 2 parallels the analytical approach employed in Study 1. Thus, presenting this analysis allows for direct methodological consistency and comparability across studies. Specifically, it enables us to assess whether the moderation observed through measured continuous variables in Study 1 replicates conceptually under experimental manipulation conditions in Study 2, thereby offering a more integrative and robust narrative across both studies.

4. Study 2 Discussion. English language still needs to be revised. The second sentence begins with “But unlike Study 1, Study 2 revealed in this study we observed…” which is not correct. Overall, once more, the whole article should be more carefully checked.

**Response:** We reviewed the entire manuscript sentence by sentence to ensure the accuracy of the language. In the track changes version of the manuscript, various edits can be seen. We believe that these revisions have made the manuscript linguistically sufficient.

5. Both in the Study 2 discussion and in the general discussion, the authors cannot state that their effect of experimentally manipulated ingroup norms is STRONG (or stronger), as they have not conducted a test on the strength of the effect(s). They should merely state that the effect is significant.

**Response:** We identified the sentences containing these expressions one by one and revised them in line with the Reviewer’s suggestions.

6. While I get the arguments by the authors, I still feel that the (long) text distinguishing between descriptive and injunctive norms has limited relevance, given that the experimental manipulation does not focus exclusively and univocally on injunctive norms. That said, I have repeated more or less the same comment three times, and it is up to the editor to decide whether this change is necessary or not.

**Response:** In this version, we believe that we briefly stated the differentiation between the two norms at the beginning of Study 2, but we are also eager to follow the Editor’s recommendation on this matter and will shorten this part of the manuscript if necessary.
